# Comment on: Endogenous retroviruses expressed in human tumours cannot be used as targets for anti-tumour vaccines

**DOI:** 10.1016/j.tranon.2021.101040

**Published:** 2021-03-30

**Authors:** Amaia Vergara Bermejo, Karen Nørgaard Nielsen, Peter Johannes Holst

**Affiliations:** 1 InProTher ApS, BioInnovation Institute, Copenhagen Bio Science Park, 2200 Copenhagen, Denmark; 2 Department of Biomedical Sciences, University of Copenhagen, 2200 Copenhagen, Denmark; 3 Center for Medical Parasitology, Department of Immunology and Microbiology, University of Copenhagen, 2200 Copenhagen, Denmark

In the short communication “Endogenous retroviruses expressed in human tumours cannot be used as targets for anti-tumour vaccines” [1] Joachim Denner argues that the idea we recently proposed [2] lacks scientific basis and may be harmful. We do not agree with these statements.

In the short communication, it is argued that animal studies have not been able to demonstrate sufficient effectiveness of endogenous retrovirus (ERV) immunotherapy against murine cancers. While passive humoral and cellular immunity against murine ERVs, as Dr. Denner notes, is capable of slowing or controlling cancer growth, vaccination has also proven efficacious in a study omitted in the short communication: We have previously shown that adenoviral vectors, known to be more potent than DNA vaccination, serve as a viable vaccination strategy against murine ERV expressing cancers [3]. Still, advancing from murine to human ERVs (HERVs) in mouse models is not necessarily straightforward. Accordingly, Dr. Denner progresses to an acknowledgement of the effectiveness of passive immunotherapy in models of human cancer in immunocompromised mice – models where for obvious reasons vaccination cannot be tested. As covered in our review, vaccination strategies have however proven effective against murine tumor cell lines genetically modified to express HERV-K. Thus, we argue that data from mouse models, including that presented by Dr. Denner, supports targeting ERVs as a viable approach for mobilizing the immune system to kill cancer cells.

The next concern presented is the safety aspect of vaccinating against an antigen that is expressed in placenta and embryonic stem cells (ESC). While the expression of HERV-K in the human placenta clearly warrants caution when considering HERV-K directed immunotherapies for women of childbearing potential, we must argue that a potential immune reaction against the placenta cannot be a sufficient argument to withhold cancer treatment for those accepting not to have further children. Patient education and oncofertility counselling are key factors here. Likewise, the expression of HERV-K in embryonic and pluripotent stem cells is not a relevant concern at a level to preclude development of new cancer therapies. Indeed, active cancer immunotherapies deliberately targeting fetal antigens have been studied for more than a century, and several of the most intensively studied cancer antigens such as MAGE-A3 and NY-ESO-1 show prominent embryonic [4] and mesenchymal [5] stem cell expression, respectively. For such antigens there have been clinical vaccine trials in phase 3 for MAGE-A3 [6] and a specific CAR-T cell therapy targeting NY-ESO1 [7] without specific safety signals. Likewise, existing ERV-specific T cell responses also points towards a favorable safety profile: HERV-K specific T cells are induced in breast [8] and ovarian cancers, [9] but unique safety signals do not appear when patients carrying these tumors respond to immunotherapy.

The final concern about immune cells expressing ERV genes is unfounded. As Dr. Denner mentions elsewhere in the short communication, expression patterns of ERVs vary greatly between species, and the studies documenting expression were carried out in murine and porcine cells. Apart from expression induced by concomitant exogenous viral infection, no data exist on HERV-K expression in human lymphocytes. Still, Dr. Denner ignores the fact that immune responses against antigens expressed on dendritic cells are a natural part of tumor immune responses and that their deliberate enhancement by vaccination is safe and effective against solid tumors [10].

In summary, we find that the evidence, including the studies highlighted in Dr. Denner's commentary, clearly supports the potential benefit of immune responses against HERV-K induced by both vaccination and passive immunotherapy. Moreover, it suggests an acceptable safety profile for the treatment of relevant cancers.

## CRediT authorship contribution statement

**Amaia Vergara Bermejo:** Investigation, Writing – original draft, Writing – review & editing. **Karen Nørgaard Nielsen:** Investigation, Writing – original draft, Writing – review & editing. **Peter Johannes Holst:** Investigation, Writing – original draft, Writing – review & editing, Funding acquisition.
